# Report of a Case of Diphtheria with Rare Complications

**Published:** 1887-02

**Authors:** J. A. Green

**Affiliations:** Georgia


### REPORT OF A CASE OF DIPHTHERIA WITH RARE COMPLICATIONS.

BY J. A. GREEN, OF GEORGIA.

June 27, 1886, was called to see a little girl, aged twenty months; very small but plump, bright child for her age ; had been reared by a wet nurse ; health good to date, except a little diarrhœa last Summer while teething. Found the patient with high fever ; temperature not taken ; pulse 135 per minute ; considerable crepitation in both lungs, and breathing 65 per minute ; also some cough. The following prescription was made :

℞. Syrup scillæ.... } aa........................ʒ ij,

Syrup ipecac... }

Tincture opii et camphor atæ................gtts xxx,

Syrup simplis, q. s. add.....................℥ ij. M.

Sig. A teaspoonful every two hours until cough is relied.

Also,

℞. Extract aconite rad, fl.......................gtts x,
Extract gelseminum, fl......................gtts xx,
Aquæ.................................℥ iij. M.

Sig. A teaspoonful every hour till fever subsides.

A powder of the following was given at bedtime :

℞. Hydgr. cum creta.........................gr. v,
Pulv. Doveri..............................gr. i. M.

Sig. Take at bedtime.

A poultice, as warm as could be borne, with a little turpentine sprinkled on one side, put in contact with the skin, was placed all over the chest and allowed to remain until the patient complained of its burning too much, then to be removed and a warm poultice without any turpentine, renewed every two or three hours.

28th, 7 a. m.—No fever ; respiration almost natural, and but very little crepitation of lungs. Had a good bilious discharge from bowels at 5 a. m., and two more during the day. Two grains of quiniæ sulph. was given every two hours till 6 grains were taken, commencing at 8 a. m. The child got up and played about the room until late in the afternoon, when fever began to rise. 7 p. m.—Temperature 102½ ; respiration as on previous night ; pulse 140 per minute ; tight, croupy cough, and quite restless. Examined throat and found the pharynx inflamed; tonsils very much enlarged, with a white membranous deposit dotted about on their surfaces and also on the base of the tongue, nearly all of which came off by mopping out the throat with a soft cotton mop saturated with spirits of turpentine ; very little crepitation of lungs, if any. Same prescription was repeated as on the night previous, except to the febrifuge mixture was added 24 gtts. of fluid extract of ipecac ; 3 grains of calomel, with 1 grain of Dovers' powder (to procure rest) given at bedtime (9 p. m.), and throat thoroughly mopped, as before stated.

29th, 1 a. m.—Temperature same as when last taken ; also the pulse and breathing. For the former fever mixture was substituted varatrum viride in 1-drop doses every hour. 4 a. m.—But little fever ; temperature 100½ ; pulse 98 per minute ; breathing 40 per minute ; resting quite easy. She was kept under the influence of the veratrum all day. Quinine in increased doses was given, and commenced a little earlier. The throat was mopped again at 8 p. m. by Dr. Mayson, a professional friend, who was called in my absence, the family becoming frightened at the nausea occasioned by the veratrum. Her fever began to rise again at 7 p. m. At 11 p. m. Dr. Mayson was called in consultation. He suggested 1 grain of calomel, a dose to be given with quinine as the day before, which suggestion was accepted, and commenced early next morning. At 12 o'clock she began to get quite hoarse.

30th, 1 a. m.—Five drops of fluid extract of ipecac, with a teaspoonful of the above cough mixture, was given, from which she vomited and expelled a quantity of diphtheritic membrane and viscid mucus ; after which, was very much relieved and rested extremely well until 10 a. m., although she had considerable fever. The throat was mopped out again in forenoon. From 1 till 11 p. m. she had no fever ; after which time the temperature rose rapidly.

By 12 m. the thermometer registered 104½ ; pulse could hardly be counted on account of extreme frequency (155 or 160 per minute). With a few drops of veratrum the fever subsided, the little sufferer becoming very sick at the stomach and vomited a good deal of bilieus matter, with more of white membrane and mucus.

July 1st, 7 a. m.—No fever ; eruption all gone ; rested easy during the night ; secretions open, and condition of throat much improved, having but little of the diphtheritic deposits in it ; tonsils lessened in size. Quinine, without the calomel, was repeated in doses somewhat less than the day previous. Took nourishment without much persuasion, when, before, it had been difficult to get her to take enough to sustain life.

July 2.—Patient pretty much the same as on yesterday ; but little fever. Quinine 2 grains was given morning, noon and night ; also nourishment was taken freely and relished.

July 3, afternoon.—Temperature ran up to 102, where it remained all night, notwithstanding veratrum, in 1-drop doses, every hour had been administered. 8 p. m.—An eruption began to make its appearance on the feet, hands and face, also about the joints—very red—the feet and hands almost the color of a boiled lobster—and raised somewhat like measles ; not so well marked in the face.

July 4th, 8 a. m.—Very little fever. Two grains of quinine was given every two hours until 10 grains were taken. 10 p. m.—No sign of an eruption anywhere about the patient, but the skin in the palms of the hands and soles of the feet are of a dark, red color. Nourishment every three hours was given during the day and two or three times during the night.

July 5th, 8 a. m.—No fever ; throat almost well. Quinine and nourishment taken as on the day before.

July 6.—Patient doing well, except a sore mouth (apthæ), for which the following was prescribed : Pulverized borax, pulverized white sugar, aa ʒ ss. M. S. Put a small quantity in mouth every three hours during the day.

July 7.—Quite an improvement in every respect ; calls for nourishment frequently, also for her doll and other playthings.

July 8.—Bowels eommenced to run off in forenoon, and had six or seven actions during the day ; no fever or any other unfavorable symptoms, except the diarrhœa, for which was prescribed :

℞. Sub-nitrate bismuth........................gr. ix,
Pulverized Doveri..........................gr. ij. M.

Divide in six powders.

S. One powder every two hours till bowels are checked.

Also, quinine in 2 grain doses three times a day, with milk punch and essence of chicken as nourishment, in small quantities.

July 9.—Bowels under control; moved twice during the day; apthæ about gone.

July 10.—Patient a little restless, with an eruption very much like flea-bites on the extremities and body, which by 2 p. m. had entirely disappeared, leaving the surface perfectly smooth and normal in color. Bowels moved four times during the day; the two first discharges were normal in character, the others dark green and a little streaked with blood. The powders of bismuth and Dovers were repeated; quinine and nourishment given as before.

July 11.—Was called at 1 a. m. to come as quickly as possible to the little sufferer. The mother said, as I entered the door, "Doctor, she has turned perfectly black all over her stomach!" I found on the breast and abdomen just as the lady had said—perfectly black (by lamp-light)—"purpura"; also a few patches on arms and face; around these patches I noticed the eruption was similar to that of the night previous, which, however, soon assumed a petechial character and quite dark in places, and finally the eruption at every place assumed a dark violet color. The bowels moved eight or ten times by 8 p. m., like the two last of the evening before. Dr. Mayson again saw the case with me. No rise of temperature; pulse frequent and weak; the patient quite restless until after a dose of the following mixture was given and the bismuth powder stopped:

℞. Tannin..............................ʒ i,

Sub. nit. bismuth..............................ʒ ss,

Act. morphine..............................gr. ij,

Vin. Port..............................℥ ij,

Aquæ..............................℥ ij. M.

Sig. Teaspoonful every two hours until bowels are checked, after which to be given after each motion.

As soon as I discovered the purpura I commenced giving 5 drops of the tincture of chlo. Ferri, with a teaspoonful of the saturated aqueous solution of chlo. pot. every four hours; also, a poultice about the same as first used over the entire abdomen. Dr. Word saw the patient with me to-day. At 12 m. temperature normal; pulse not so frequent, and resting apparently better than in the forenoon; bowels moved three or four times from this time till 1 a. m. the following morning; the discharges very dark, almost black, from the medicine; purpura remained the same for that day. Medicine given as prescribed; also stimulants given pretty freely in the forenoon and occasionally in the afternoon.

July 12.—The patient began to grow worse at 4 a. m. ; pulse almost imperceptible at times, but came up quickly after administering whisky ; temperature normal ; bowels moved frequently until 11 a. m., and containing more blood than the day before ; in the afternoon the bowels moved three times, with no mucus—only a little bloody water. At Dr. Word's suggestion the day before, 4 drops of the elixir of vitriol was given every four hours, alternated with the iron mixture ; imperial granum with sweet milk, every three hours, alternated with chicken essence ; also whisky every half hour, a teaspoonful as strong as could be borne by patient. Several new patches appeared about 1 p. m.

July 13.—Patient restless all night ; pulse frequent and feeble ; temperature normal until about 5 a. m., when hands and feet began to get cold. At 4 a. m. the hands and feet turned very red (scarlet), which passed off in a litt e while, leaving the purpuric condition as on the body. Eight a. m.—No pulsation of the radial arteries ; arms cold to armpits, and legs cold above knees ; temperature 97 ; palor of countenance. Hot flannels and sinapism were applied to the extremities, and kept up for an hour, when the temperature was found to be as high as 101 ; pulse again perceptible and, for the first time during her illness sweated profusely around the neck, face and head. The artificial heat was kept up until death at 1 p. m.

I allowed no chi dren in the room, as soon as I found the throat involved. There were two other children in the house all the time before the patient was taken and after its death ; to date have had no symptoms of diphtheria. Antiseptics were freely used about the house from the commencement to the termination of the case.

We have thus described, in detail, a case of diphtheria presenting peculiar and somewhat unusual features. If any professional brother has met with a similar case I hope that the same, with the treatment, will be reported in the RECORD.

